# Inhibition of galectin-3 ameliorates the consequences of cardiac lipotoxicity in a rat model of diet-induced obesity

**DOI:** 10.1242/dmm.032086

**Published:** 2018-02-01

**Authors:** Gema Marín-Royo, Isabel Gallardo, Ernesto Martínez-Martínez, Beatriz Gutiérrez, Raquel Jurado-López, Natalia López-Andrés, Josué Gutiérrez-Tenorio, Eduardo Rial, María Visitación Bartolomé, María Luisa Nieto, Victoria Cachofeiro

**Affiliations:** 1 Departamento de Fisiología, Facultad de Medicina, Universidad Complutense de Madrid and Instituto de Investigación Sanitaria Gregorio Marañón (IiSGM), Madrid 28040, Spain; 2 Instituto de Biología y Genética Molecular, CSIC-Universidad de Valladolid, Valladolid 47003, Spain; 3 Cardiovascular Translational Research, Navarrabiomed (Miguel Servet Foundation), Instituto de Investigación Sanitaria de Navarra (IdiSNA), Pamplona 31008, Spain; 4 Centro de Investigaciones Biológicas, CSIC, Madrid 28040, Spain; 5 Departmento de Oftalmología y Otorrinolaringología, Facultad de Psicología, Universidad Complutense, Madrid 28223, Spain; 6 Ciber de Enfermedades Cardiovasculares (CIBERCV). Instituto de Salud Carlos III, Madrid 28029, Spain

**Keywords:** Galectin-3, Insulin resistance, Lipotoxicity, Mitochondria, Obesity, Oxidative stress

## Abstract

Obesity is accompanied by metabolic alterations characterized by insulin resistance and cardiac lipotoxicity. Galectin-3 (Gal-3) induces cardiac inflammation and fibrosis in the context of obesity; however, its role in the metabolic consequences of obesity is not totally established. We have investigated the potential role of Gal-3 in the cardiac metabolic disturbances associated with obesity. In addition, we have explored whether this participation is, at least partially, acting on mitochondrial damage. Gal-3 inhibition in rats that were fed a high-fat diet (HFD) for 6 weeks with modified citrus pectin (MCP; 100 mg/kg/day) attenuated the increase in cardiac levels of total triglyceride (TG). MCP treatment also prevented the increase in cardiac protein levels of carnitine palmitoyl transferase IA, mitofusin 1, and mitochondrial complexes I and II, reactive oxygen species accumulation and decrease in those of complex V but did not affect the reduction in ^18^F-fluorodeoxyglucose uptake observed in HFD rats. The exposure of cardiac myoblasts (H9c2) to palmitic acid increased the rate of respiration, mainly due to an increase in the proton leak, glycolysis, oxidative stress, β-oxidation and reduced mitochondrial membrane potential. Inhibition of Gal-3 activity was unable to affect these changes. Our findings indicate that Gal-3 inhibition attenuates some of the consequences of cardiac lipotoxicity induced by a HFD since it reduced TG and lysophosphatidyl choline (LPC) levels. These reductions were accompanied by amelioration of the mitochondrial damage observed in HFD rats, although no improvement was observed regarding insulin resistance. These findings increase the interest for Gal-3 as a potential new target for therapeutic intervention to prevent obesity-associated cardiac lipotoxicity and subsequent mitochondrial dysfunction**.**

## Abstract (precis)

**Summary:** Inhibition of Gal-3 activity reduced the excessive cardiac accumulation of lipids in rats fed a high fat diet. This was accompanied by the amelioration of mitochondria damage observed in obese rats.

## INTRODUCTION

Obesity is a chronic disease characterized by excessive accumulation of adipose tissue and lipids forming ectopic fat deposits in different tissues, including the heart (Abdurrachim et al., 2014; French et al., 2016; Ghosh et al., 2011). This excessive accumulation of lipid in the heart – termed cardiac lipotoxicity – can trigger cellular alterations since lipids are important regulators of cardiac function through their role in membrane structure, transport, signaling and as substrate for β-oxidation for obtaining energy in the mitochondria (Cedars et al., 2009; Lim et al., 2011). Cardiac lipotoxicity not only involves an excessive accumulation of intra-myocellular triglycerides (TGs) in the heart but also changes in different lipid classes, as well as in their fatty acid composition, thereby facilitating the formation of active lipid mediators which affect metabolism and cardiac function, in part by altering mitochondrial function (Bugger and Abel, 2008; Elezaby et al., 2015; Lucas et al., 2016; Wang et al., 2015).

A common additional feature of the obese heart is impaired insulin signaling, which represents an adaptation of the heart to an excess of calories, which promotes the development of diabetic cardiomyopathy (Guo and Guo, 2017; Jia et al., 2016; Riehle and Abel, 2016). This condition not only alters cardiac metabolism but also increases myocardial oxygen consumption, reduces cardiac efficiency by affecting mitochondrial function and increases oxidative stress with the mitochondria being the main source of reactive oxygen species (ROS) (Boudina et al., 2007; Elezaby et al., 2015; McMurray et al., 2016).

Galectin-3 (Gal-3) is a member of a β-galactoside-binding lectin family produced in the heart and whose expression is upregulated in obesity (Martinez-Martinez et al., 2014). Its role as a central mediator of cardiovascular fibrosis and the inflammatory processes present in different pathological situations, including obesity, has been demonstrated (Martinez-Martinez et al., 2014, 2015b). In addition, the potential role of Gal-3 as a regulator of cardiac oxidative stress which can facilitate the development of fibrosis has been suggested since it is able to upregulate Nox4 expression in cardiac fibroblasts (He et al., 2017). In addition, we have reported Gal-3 to be a mediator of leptin-induced ROS production in the heart of obese rats (Martinez-Martinez et al., 2014, 2015b). However, information regarding the role of Gal-3 in the metabolic consequences of obesity is not well established since it has exhibited roles of being both mediator and preventer of metabolic disorders (Martinez-Martinez et al., 2016; Menini et al., 2016). Therefore, the aim of this study was to explore the potential contribution of Gal-3 to the metabolic disturbances associated with obesity. In addition, we have explored whether its participation is, at least partially, acting on mitochondrial damage. To address this issue, we analyzed the effect of the specific Gal-3 inhibitor modified citrus pectin (MCP) (Martinez-Martinez et al., 2015b) by using an animal model of diet-induced obesity and cultured cardiomyoblasts stimulated by palmitic acid.

## RESULTS

### Consequences of Gal-3 activity inhibition on body weight, cardiac function and fibrosis, and blood pressure in rats that had been fed a high-fat diet

Animals that are fed a high-fat diet (HFD) show an increase in body weight, cardiac hypertrophy and interstitial fibrosis, although no changes in cardiac function or blood pressure, when compared to control rats (Martinez-Martinez et al., 2015b) after 6 weeks of HFD intake. Gal-3 protein expression was upregulated in hearts of HFD rats, and MCP treatment reduced the increase in total cardiac collagen content without modifying either body weight or cardiac hypertrophy (Martinez-Martinez et al., 2015b). MCP did not affect any of these parameters in control animals (Martinez-Martinez et al., 2015b). Therefore, and to simplify the data, only rats fed on a standard diet (CT), and on HFD or HFD+MCP are presented in the results.

### Effect of Gal-3 activity inhibition on cardiac glucose use and insulin resistance in HFD-fed rats

**Fig. 1.**
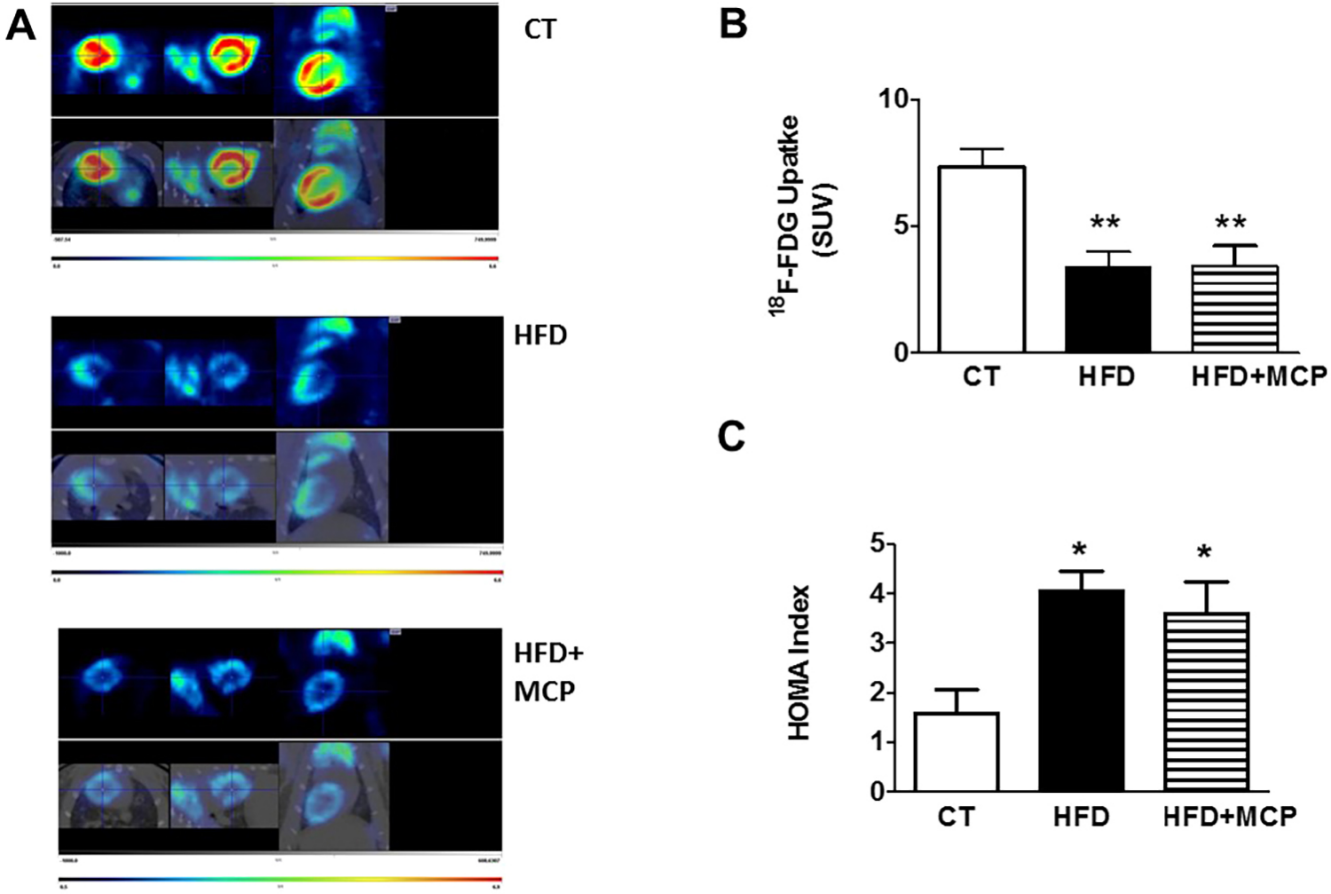
**Impact of Gal-3 inhibition on cardiac ^18^F-FDG-uptake and the HOMA index in obese rats.** (A) Representative photographs of ^18^F-Fluorodeoxyglucose (FDG) PET/CT scans of the heart from rats fed a standard diet (CT) or a high fat diet (HFD) treated with vehicle or with MCP, the inhibitor of Gal-3 activity (100 mg/kg/day) in coronal, sagittal and *trans*-axial views scaled to SUV. (B) Quantification in SUV units. (C) HOMA index of the different experimental groups. Bar graphs represent the mean± s.e.m. of 6-8 animals. *P<0.05; **P<0.01 control group.

Next, we addressed whether upregulation of Gal-3 is involved in the changes of glucose use observed in obese rats. Therefore, PET studies were performed to assess their use of cardiac glucose. Representative examples of PET images (Fig. 1A,B) revealed that uptake of fluorine-18 (^18^F)-tagged fluorodeoxyglucose (FDG) in the heart was higher in the control group that in obese animals. Administration of MCP did not increase myocardial ^18^F-FDG uptake. Similarly, dietary addition of MCP did not affect the increase of the homeostatic model assessment (HOMA) index in obese rats (Fig. 1C).

### Consequences of Gal-3 activity inhibition on cardiac lipid profile in HFD-fed rats

**Fig. 2.**
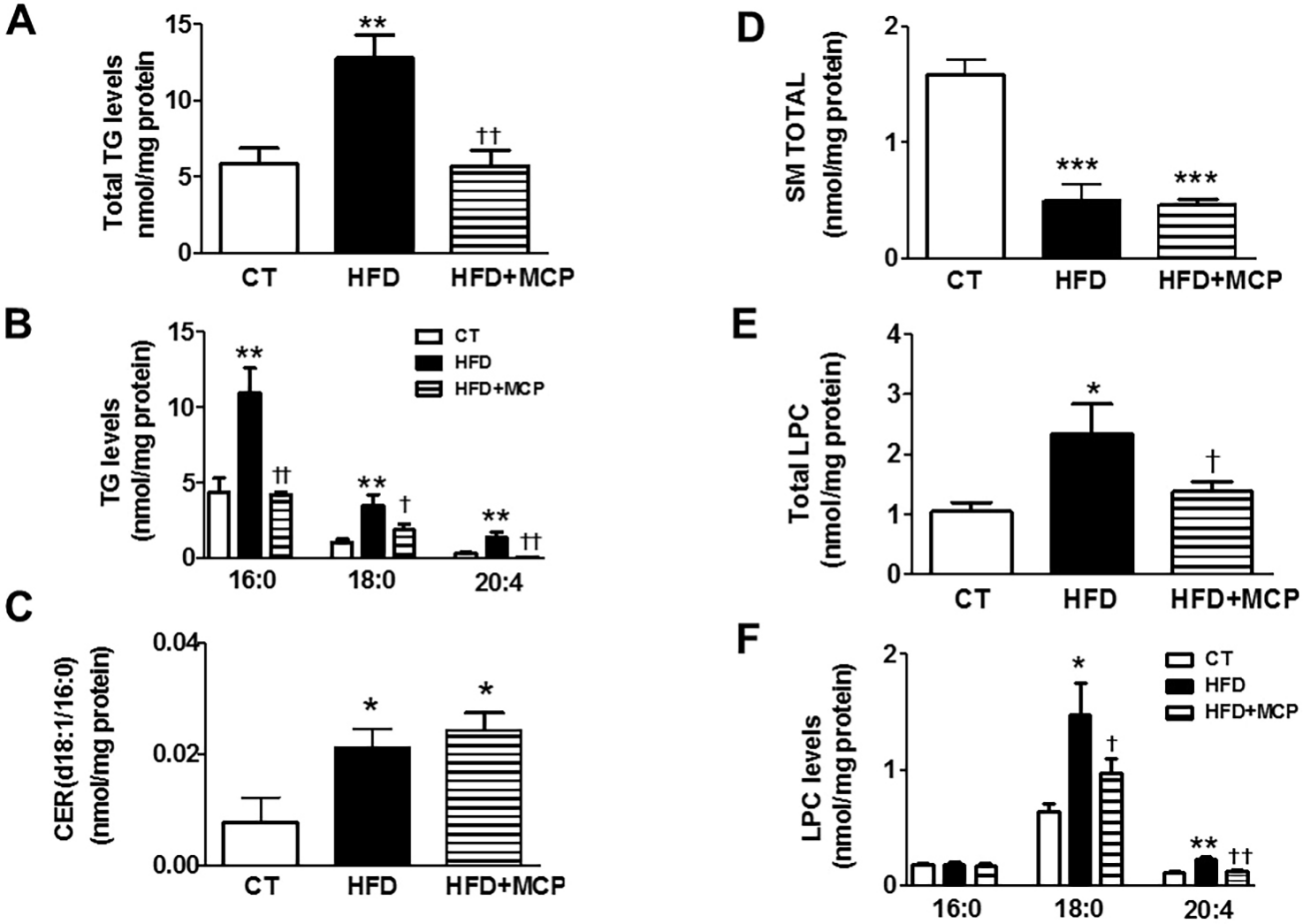
**Effects of Gal-3 inhibition on lipid species in heart from control and obese rats.** Rats were fed a standard diet (CT) or a high fat diet (HFD) and treated with control vehicle or with the Gal-3 activity inhibitor MCP (100 mg/kg/day). Cardiac levels of (A) total TGs, (B) the three main types of TG, (C) ceramide (Cer), (D) total sphingomyelins (SM), (E) total lyso phosphatidylcholine (LPC), (F) the three main types of LPC. Bar graphs represent the mean±s.e.m. of 6-8 animals.*P<0.05; **P<0.01; ***P<0.001 vs control group. ^†^*P*<0.05; ^††^*P*<0.01; ^†††^*P*<0.001 vs HFD group.

**Fig. 3.**
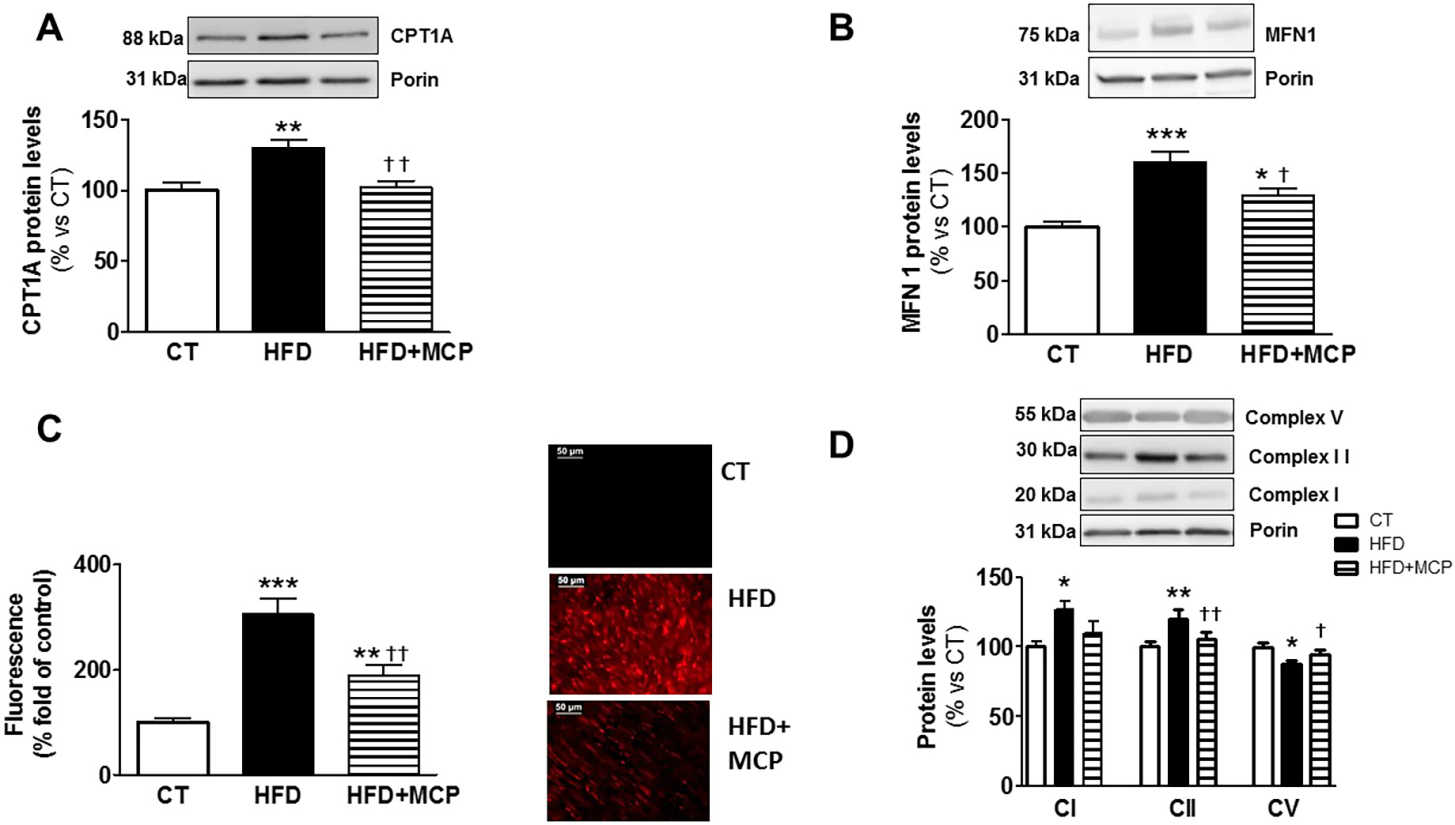
**Impact of Gal-3 inhibition on proteins and superoxide anion production in hearts from control and obese rats.** Hearts from rats fed a standard diet (CT) or a high fat diet (HFD) treated with vehicle or with the inhibitor of Gal-3 activity (modified citrus pectin; MCP; 100 mg/kg/day) were analyzed. Protein expression of (A) carnitine palmitoyl transferase IA (CPT1A), (B) mitofusin 1 (MFN1), (D) for mitochondrial complexes I (subunit NDUFB8), II (30 kDa) and V (alpha subunit) are presented. (C) Representative microphotographs (magnification ×40) of cardiac sections labeled with MitoSOX and quantification of superoxide anions in heart. Bar graphs represent the mean±s.e.m. of 6-8 animals normalized to porin. Scale bars: 50 µm. *P<0.05; **P<0.01 vs control group. ^†^*P*<0.05; ^††^*P*<0.01 vs HFD group.

As expected, obese animals showed an increase in total TG content in the heart (Fig. 2A), mainly due to an accumulation of TGs enriched with palmitic acid (16:0) and, to a lesser extent, TGs enriched with stearic acid (18:0) and arachidonic acid (20:4) (Fig. 2B). In fact, obese animals showed an overall increase (4-fold; *P*<0.001) in TGs enriched with saturated fatty acids, although no significant changes were observed in the amounts of polyunsaturated fatty acids (data not shown). Treatment with MCP was able to reduce this rise observed in animals that were fed an HFD (Fig. 2A-B). An increase in the only type of ceramide (Cer) detected (Cer d18:1/16:0) was observed in HFD rats as compared with control animals (Fig. 2C). However, a reduction in the levels of total sphingomyelin (SM) was observed in HFD-fed animals as compared with control animals (Fig. 2D), a consequence of the decrease in the majority of the eight species of SM detected. MCP treatment was unable to revert this SM reduction (Fig. 2C,D). A negative correlation was observed between levels of total SM and Cer (r=−0.5274; *P*=0.0433). In addition, a correlation was found between Cer and ^18^F-FDG cardiac uptake levels (r=−0.668; *P*=0.0065) and also between total SM levels and those of ^18^F-FDG cardiac uptake (r=0.6220; *P*=0.0175). A similar correlation was found between the HOMA index and Cer levels (r=0.6299; *P*=0.012). Given that phosphatidyl choline (PC) and lysophosphatidyl choline (LPC) are involved in various diseases and because altered levels of LPC in the serum is considered to be a specific metabolic trait associated with obesity (Li et al., 2014; Tulipani et al., 2016), we focused on the types of PC and LPC found in the hearts of HFD rats. No significant differences were observed in the levels of total PC in the hearts of CT and HFD rats. About 56 types of PC were detected in hearts from CT rats, and their expression pattern was very similar to those observed in the heart of the HFD group (data not shown). However, an increase was found in the level of total LPC in HFD compared to CT animals (Fig. 2E). This increase was mainly due to the rise in those types of LPC enriched with stearic acid – LPC (18:0), or arachidonic acid – LPC (20:4) (Fig. 2F). The levels of detected LPC (16:0), LPC (18:2), LPC (18:3), LPC (22:5) and LPC (22:6) were not affected by the HFD. Treatment with MCP was able to prevent the increase in total LPC levels and in those enriched with stearic (18:0) or arachidonic (20:4) acids in HFD rats (Fig. 2E,F). Total LPC levels were correlated to those TGs enriched with palmitic acid (r=0.6841; *P*=0.0048). The increase in TGs was associated with higher levels of CPT1A, the enzyme that controls the entry of long-chain fatty acyl CoA into mitochondria (Fig. 3A). Treatment with the inhibitor of Gal-3 was able to normalize CPT1A levels in HFD rats (Fig. 3A).

### Consequences of inhibition of Gal-3 activity on cardiac mitochondria dynamic in HFD-fed rats

Considering the accumulation of fatty acids in the mitochondria of obese animals, we explored the consequences of this accumulation on the mitochondrial dynamics. To this end, we evaluated the levels of two proteins, mitofusin 1 and DRP1, involved in the process of fusion and fission. As shown in Fig. 3B, the protein levels of mitofusin 1 are higher in obese animals than in controls. MCP treatment was able to reduce these high levels. By contrast, levels of the fission marker DRP1 were unaffected by either obesity or Gal-3 inhibition (data not shown). In addition, obese animals showed an increase in mitochondrial oxidative stress in response to MitoSOX (red mitochondrial superoxide indicator) as indicated by more-intense fluorescence staining in the heart of the obese animals compared with control animals (Fig. 3C). A correlation was found between levels of mitochondrial ROS, and levels of total TGs (Table 1) and of TGs enriched with stearic or arachidonic acid (Table 1). We also evaluated the protein levels of the components of mitochondrial respiratory chain complexes. As shown in Fig. 3D, obesity exerts a different impact on different complexes since obese animals show an increase in complexes I and II, and a decrease in complex V, the ATP synthase. No changes were observed in the levels of complexes III and IV (Fig. S1). MCP treatment was able to reverse these changes.

**Table 1.**
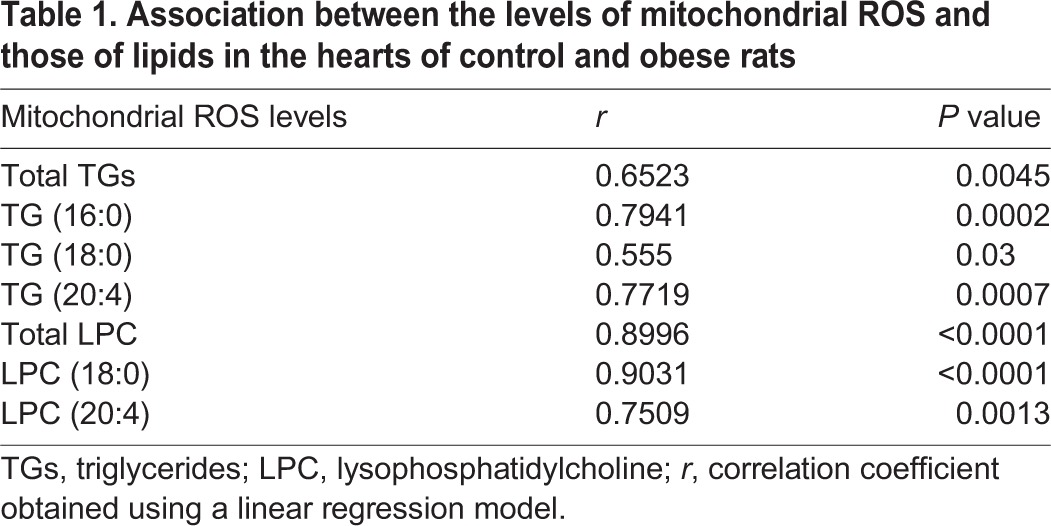
**Association between the levels of mitochondrial ROS and those of lipids in the hearts of control and obese rats**

### Effect of palmitic acid on mitochondrial function in rat cardiomyoblasts – consequences of Gal-3 activity inhibition

**Fig. 4.**
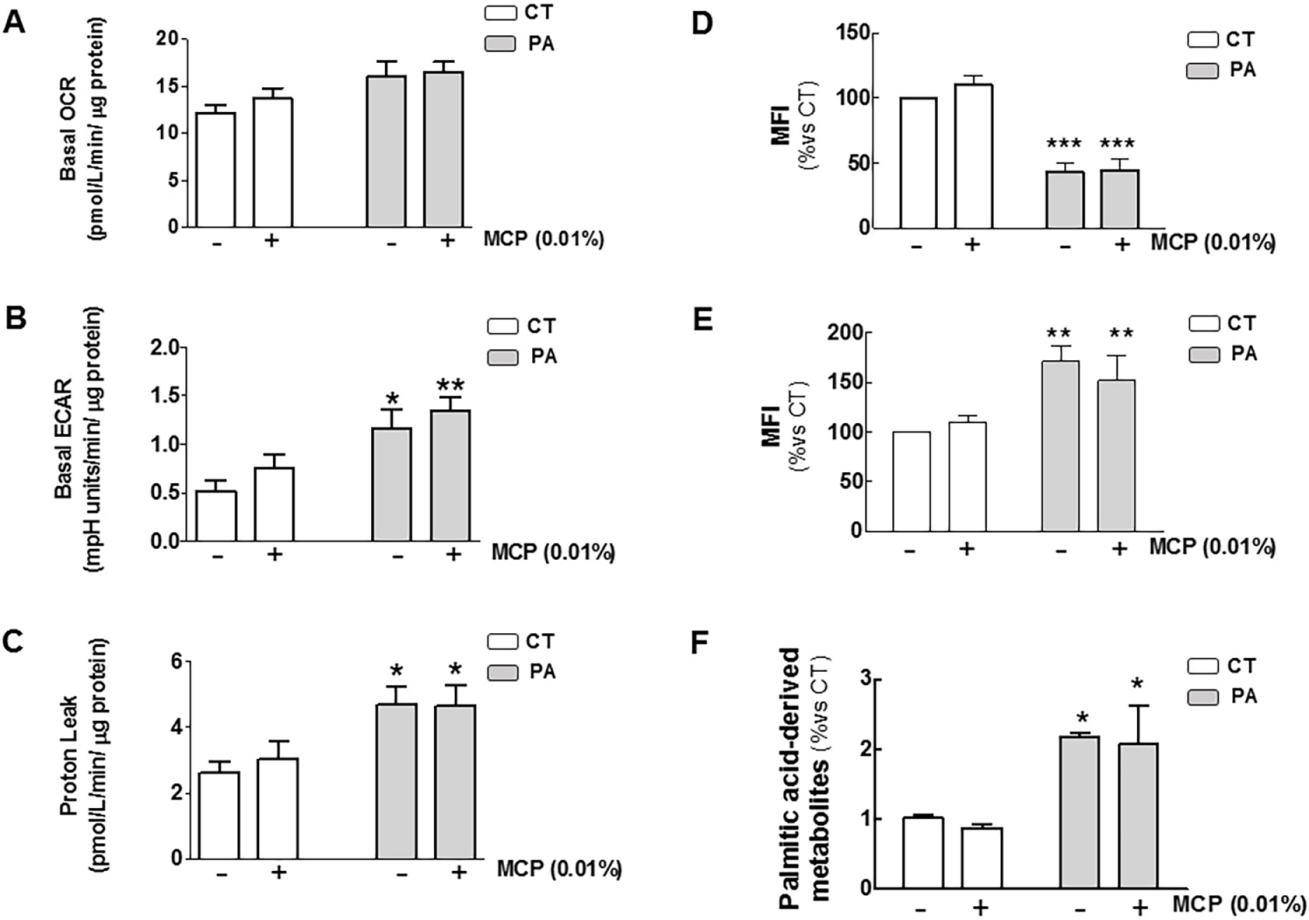
**Effects of Gal-3 inhibition on mitochondrial function, glycolysis, membrane potential, ROS production and β-oxidation in palmitic-acid-treated H9c2 cells.** (A) Basal respiration expressed as oxygen consumption rate (OCR). (B) Basal glycolysis expressed as extracellular acidification rate (ECAR). (C) Proton leak respiration expressed as OCR. (D) Quantification of flow cytometry analysis of mitochondrial membrane potential in cells stained with Rhodamine 123 expressed as mean fluorescence intensity (MFI). (E) Quantification of flow cytometry analysis of mitochondrial superoxide anions in cells labeled with MitoSOX expressed as MFI, (F) Quantification of β-oxidation in cardiac myoblasts treated for 24 h with palmitic acid (200 µmol/l) in the presence of absence inhibitor of Gal-3 activity (modified citrus pectin; MCP; 0.01%). Error bars represent the mean±s.e.m. of four assays. **P<0.01; ***P<0.001 vs vehicle-treated cells (CT).

Taking the observed accumulation of palmitic acid into consideration, we decided to explore its effects on mitochondrial function by using cultured rat cardiomyoblasts. Fig. S2 shows that palmitic acid was unable to affect cell viability at the doses used (100 μmol/l, 200 μmol/l and 300 μmol/l). Next, we performed a ‘mitochondrial stress test’ in order to evaluate the mitochondrial bioenergetics caused by palmitic acid. The general scheme of the stress test is shown in Fig. S3A. Palmitic acid caused a modest and dose-dependent increase in the basal rate of respiration (Fig. S3A) and was paralleled by a dose-dependent increase of the extracellular acidification rate (ECAR) signal, indicating that palmitic acid is lowering the OXPHOS efficiency due to its uncoupling capabilities (Fig. S3B). Indeed, the proton leakage rate (Fig. S3C) was significantly increased in the palmitic acid-treated cells. The presence of MCP did not significantly alter the cellular energetics induced by palmitic acid (200 µmol/l; Fig. 4A-C). Additionally, as shown in Fig. S4A,B, the presence of palmitic acid for 24 h was able to reduce Rhodamine 123 staining in rat cardiomyoblasts in a dose-dependent manner, thereby reinforcing the idea that the increase in proton leak due to fatty acid uncoupling causes a reduction in the mitochondrial potential membrane. The presence of MCP did not alter the decrease in Rhodamine 123 staining caused by palmitic acid (200 µmol/l; Fig. 4D). Likewise, the presence of triacsin C – the inhibitor of acyl-CoA synthase – was unable to modify the effect induced by palmitic acid (Fig. S4C). In addition, palmitic acid was able to increase mitochondrial ROS production in a dose-dependent manner, as suggested by an increase in MitoSOX-induced fluorescence in palmitic acid-treated cells relative to that of vehicle-treated cells (Fig. S5A-B). The presence of MCP did not significantly alter the fluorescence intensity induced by MitoSOX in cells treated with palmitic acid (200 µmol/l; Fig. 4E). This was accompanied by a dose-dependent reduction in staining with the fluorochrome 10-N-nonyl-Acridin Orange (NAO), indicating an increase in the levels of oxidized cardiolipins (Fig. S6). Finally, palmitic acid was able to increase the β-oxidation in a time-dependent manner (Fig. S7). The presence of MCP did not significantly alter this effect induced by palmitic acid (Fig. 4F).

## DISCUSSION

The role of Gal-3 as a central mediator of cardiovascular fibrosis and the inflammatory processes present in different pathological situations has been amply demonstrated (Calvier et al., 2013, 2015; Martinez-Martinez et al., 2014, 2015a,b). We here report for the first time that Gal-3 can modulate some of the metabolic consequences of obesity, since the inhibitor of Gal-3 activity MCP reduced cardiac lipotoxicity and ameliorated the mitochondrial damage observed in the heart of obese rats.

Our present data show a significant increase in TG levels in the heart of normotensive obese animals, confirming previous clinical and experimental studies (Kroon et al., 2017; Shimabukuro et al., 2013). This increase was mainly a consequence of the rise in the levels of TGs enriched with saturated (16:0, 18:0) and polyunsaturated (20:4) fatty acids. We have also found an increase in LPC levels, mainly of those enriched with fatty acids 20:4 and 18:0. A similar increase has been reported for circulating LPC levels in obese patients and in experimental models of obesity (Eisinger et al., 2014; Li et al., 2014; Tonks et al., 2016). It should be noted, however, that reductions have been reported in other studies, which may be the result of varying LPC fatty acid composition (Li et al., 2014; Tulipani et al., 2016; Wahl et al., 2012) and suggests a more-complex effect of obesity in this lipid class. In contrast, PC profiles were not affected in HFD rats. Therefore, our data support the idea that cardiac lipotoxicity involves not only variations in the type of lipid but is also due to differences in their fatty acid composition.

Neither TG nor LPC levels seem to be major determinants of the altered cardiac glucose use observed in HFD animals because no correlation was found amongst these parameters. In addition, treatment with MCP was able to normalize both cardiac TG and LPC levels without altering the abnormal ^18^F-FDG cardiac uptake. These data confirm previous observations that found no link between either TG or LPC circulating levels and insulin resistance in the context of obesity (Coen and Goodpaster, 2012; del Bas et al., 2016; Eisinger et al., 2014; Tulipani et al., 2016). However, the reduced cardiac SM levels observed in HFD rats might participate in the cardiac insulin resistance observed in these animals since a direct correlation between levels of SM and ^18^F-FDG uptake was observed, confirming previous data (Denimal et al., 2016; Tonks et al., 2016). In agreement with this observation, we have found that cardiac SM levels are independent predictors of GLUT4 cardiac levels in HFD (Marin-Royo et al., 2017). In addition, and considering the role of Cer in the pathogenesis of diabetes (Galadari et al., 2013), our data here support the participation of this lipid class, whose levels were increased in response to a HFD and associated with increased cardiac levels of ^18^F-FDG uptake. Cer levels result from both *de novo* synthesis as well as hydrolysis of SM, suggesting a link between both lipids; in fact, a negative correlation was found between them. A variety of potential mechanisms – oxidative stress, changes in mitochondrial function and endoplasmic reticulum stress – might underlie these effects (Fucho et al., 2017; Petersen and Shulman, 2017; Yazici and Sezer, 2017).

Our present study shows an increase of mitochondrial oxidative stress in the heart of normotensive obese animals, which was accompanied by some mitochondrial alterations: an increase in CPT1A, mitofusin 1, and respiratory chain complexes I and II, as well as a reduction of complex V. These alterations suggest that changes occur not only in the context of mitochondrial machinery but also in that of mitochondrial morphology. This is in agreement with the concept that mitochondrial dysfunction is one mechanism that participates in the cardiac damage associated with obesity, as mitochondria play a central role in the energy production essential in maintaining cardiac activity (Mercer et al., 2010; Wang et al., 2015). The fact that treatment with MCP reduced oxidative stress and normalized the levels of CPT1A, mitofusin 1 and respiratory chain complexes further supports this role. In fact, connections between oxidative stress, lipotocixity and mitochondrial dysfunction has been suggested (Mercer et al., 2010; Schulze et al., 2016; Wang et al., 2015). Supporting this concept, we have found a correlation between the cardiac levels of TGs and LPC, and those of mitochondrial ROS in MCP-treated and untreated HFD rats. In addition, we have observed that palmitic acid, the most elevated fatty acid in TGs (the main cardiac energetic reservoir of HFD rats) was able to stimulate mitochondrial ROS production in H9c2 cells, confirming previous observations (Miller et al., 2005).

An increase in ROS can be the consequence of either an increase in oxidative metabolism or a reduction in antioxidant capacity (Cheng et al., 2017; Vakifahmetoglu-Norberg et al., 2017). Apart from the main contributors to mitochondrial ROS production, complex I and complex III, several oxidoreductases located in mitochondrial membrane can produce superoxide at significant rates during oxidation of fatty acids (Andreyev et al., 2015; Brand, 2010). This oxidant environment can disturb mitochondrial membrane phospholipids, including cardiolipins, as evident by the significant reduction in NAO fluorescence. The peroxidized cardiolipin generated changes in the physico-chemical properties of the mitochondrial membrane that, in turn, could be altering mitochondrial bioenergetics since cardiolipins play a central role in normal function and structure of the inner mitochondrial membrane (Birk et al., 2014; Paradies et al., 2014). In fact, this could explain the observed increase in mitofusin 1, which suggests an increase in mitofusion, a process that represents an adaptive pro-survival response against stress (Tondera et al., 2009).

The increase in the proton leak in H9c2 cells stimulated by palmitic acid suggests a reduced efficiency of the oxidative phosporylation. The increase observed in the β-oxidation of H9c2 cells in the same conditions could be explained as a compensatory mechanism for the oxidative phosphorylation reduction. This process might occur in the heart of obese animals since the decreased ATP synthase levels observed in these animals was accompanied by an increase in CPT1A involved in the mitochondrial uptake of fatty acids, an essential step for the β-oxidation in the mitochondria. However, the compensatory increase in glycolysis induced by palmitic acid in H9c2 in order to maintain ATP levels are adequate to meet the energy demands of the cell, although anaerobic ATP production might be limited in obese animals because the glucose uptake of the heart is reduced. We found that palmitic acid was able to reduce mitochondrial membrane potential independently of its transformation into acyl-CoA because inhibition of triacsin C, the enzyme involved in this step, had no effect. Indeed, it has previously been reported that fatty acids have a protonophoric activity that are likely to be mediated by the adenine nucleotide translocator (ANT) (Ash and Merry, 2011).

Our data show that the Gal-3 activity inhibitor is able to prevent some of the changes observed in the cardiac lipid profile in obese animals and, thus, supports a role of Gal-3 in cardiac lipotoxicity. In fact, addition of MCP was able to reduce the excessive accumulation of TGs and LPC. The exact mechanism through which Gal-3 participates in cardiac lipotoxicity is unclear, although its ability to produce an oxidant environment that can, finally, affect mitochondrial activity, might be an option. In support of this concept is the observation that Gal-3 colocalizes with ATP synthase in the inner membrane of mitochondria and has an inhibitory effect on ATP synthase in human colon cancer cells (Kim et al., 2008). Interestingly, MCP does not protect against palmitic acid-induced mitochondrial dysfunction in H9c2 cells *in vitro*, thus, suggesting that the beneficial effect induced by MCP in obese animals is a consequence of Gal-3 inhibition and not that of other actions of this drug, since Gal-3 production was not induced by palmitic acid being present in the cells (data not shown).

By contrast, MCP did not affect glucose homeostasis. In fact, MCP did not modify the levels of SM and Cer – that are related to insulin resistance – further supporting the lack of effect Gal-3 has in glucose homeostasis in our model. By contrast, a recent study has shown that, in mice, administration of Gal-3 is associated with insulin resistance, and that Gal-3 inhibition, pharmacologically or genetically, causes glucose intolerance in HFD animals for 8 weeks (Li et al., 2016). However, this role has not been supported by previous data that have shown that Gal-3 knockout mice that had been fed a HDF for 12 or 18 weeks present a dysregulated glucose metabolism (Pang et al., 2013; Pejnovic et al., 2013). Therefore, the specific role of Gal-3 as a player in metabolic disorders needs further investigation.

In summary, to understand the mechanisms that underlying the damage associated with cardiac lipotoxicity in the context of obesity is crucial. Our present data suggest a role of Gal-3 in this damage, draw a more-complex scenario for the actions of Gal-3 that, in obese animals, is overexpressed in the heart, and seems to modulate some of the consequences of cardiac lipotoxicity. However, further work to demonstrate its specific role in mitochondrial function will help to clarify the underlying mechanism and might also come up with new approaches in the management of obesity-related heart damage.

## MATERIALS AND METHODS

### Animal model

Male Wistar rats of 150 g (Harlan Ibérica, Barcelona, Spain) were fed either a high-fat diet (HFD) containing 35% fat [Envigo (formerly Harlan) Teklad global rodent diet number, TD.03307, MN; *n*=16] or a standard diet (CT) containing 3.5% fat (Harlan Teklad number, TD.2014, MN; *n*=16) for 6 weeks. For the same period the Gal-3 activity inhibitor MCP (100 mg/kg per day), was supplied to half of each group in the drinking water. The Animal Care and Use Committee of Universidad Complutense de Madrid approved all experimental procedures according to the Spanish Policy for Animal Protection RD53/2013, which meets the European Union Directive 2010/63/UE.

### *In vivo* PET-CT imaging to study uptake of ^18^F-fluorodeoxyglucose in the heart

Myocardial metabolic activity was evaluated by means of a small-animal dedicated dual scanner (Albira PET/CT scanner, Bruker NMI, Valencia Spain). One week before the end of the experiment, animals were starved for 18 h, followed by intraperitoneal injection with ^18^F-flurodeoxyglucose [FDG; 12.99±0.04 MBq in 0.2 ml NaCl (0.9%); Instituto Tecnológico PET, Madrid, Spain]. Twenty min later, rats underwent PET and computed tomography (CT) scanning under isoflurane anesthesia. The acquired PET and CT images were then reconstructed by, respectively, using maximum-likelihood (ML) expectation-maximization (EM) [ML-EM] and filtered-back projection algorithms. In order to account for the weight difference in rats and the [^18^F] doses of injected FDG, we calculated the standardized uptake value (SUV). The semi-quantitative SUV measurement is the most widely used in [^18^F] FDG PET studies of both small animals and humans (Byrnes et al., 2014; Deleye et al., 2014). The software used was PMOD 3.6 (PMOD Technologies Ltd., Zurich, Switzerland).

To quantify the metabolic activity the CT image of the heart from each animal was registered to its corresponding PET image. Then a three-dimensional region of interest (ROI) was drawn on the fused PET/CT image to measure the metabolic activity of the entire left ventricle. These steps were carried out by using PMOD 3.0. SUV was obtained as an index of regional metabolic activity. The SUV parameter was calculated as a ratio of the ROI radioactivity concentration (kBq/ml) measured by the scanner and the administered dose (kBq) was decay-corrected at the time of the injection, divided by the body weight (g).

### Isolation of cardiac mitochondria

Cardiac mitochondria were isolated as reported previously (Doerrier et al., 2016). Frozen hearts were placed into and washed with cold homogenization medium containing 0.075 mol/l sucrose, 1 mmol/l EDTA, 10 mmol/l Tris–HCl pH 7.4. Briefly, heart tissue was homogenized (1:10 w/v) at 800 rpm in a homogenizer (T 10 basic Ultra-turrax, Ika-Werke; Germany). The homogenates were centrifuged at 1300 ***g*** for 5 min at 4°C to remove nuclei and debris. Supernatants were separated and centrifuged at 12,000 ***g*** for 10 min at 4°C. The resulting pellets were suspended in homogenization medium and centrifuged twice at 14,400 ***g*** for 3 min at 4°C to wash the mitochondrial fraction. Mitochondrial pellets were stored at −80°C until use. Protein concentration was determined using the Bradford method.

### Western blotting

Mitochondrial proteins were separated by SDS polyacrylamide electrophoresis (PAGE) and transferred to Hybond-c Extra nitrocellulose membranes (Hybond-P; Amersham Biosciences, Piscataway, NJ). Membranes were probed with primary antibody against the mitochondrial complex (Mitoprofile Total OXPHOS – CI subunit NDUFB8, CII-30 kDa, CIII-Core protein 2, CIV subunit I and the CV alpha subunit, ab110413, Abcam, Cambridge, UK; dilution 1/1000), carnitine palmitoyl transferase I (CPT1A, ab128568, Abcam, Cambridge; dilution 1/1000), dynamin-1-like protein (DRP1, ab56788, Abcam, Cambridge; dilution 1/1000), mitofusin 1 (MFN1, ab57602, Abcam, Cambridge; dilution 1/1000) and porin (ab15895, Abcam, Cambridge; dilution 1/1000) as a mitochondrial protein loading control. Signals were detected using the ECL system (Amersham Pharmacia Biotech). Results are expressed as an n-fold increase over the values of the control group in densitometric arbitrary units.

### Measurement of mitochondrial ROS production

For detection of mitochondrial ion O_2_^−^ production, cardiac sections (6 µm) were incubated with MitoSOX^TM^ (red mitochondrial superoxide indicator; 5 µmol/l) for 10 min at 37°C. MitoSOX Red reagent is a live-cell permeant probe and is rapidly and selectively targeted to the mitochondria. Once in the mitochondria, MitoSOX Red reagent is oxidized by superoxide and exhibits red fluorescence. MitoSOX Red reagent is readily oxidized by superoxide but not by other reactive oxygen species (ROS) – or reactive nitrogen species (RNS) – generating systems. The oxidation product becomes highly fluorescent upon binding to nucleic acids. Fluorescent signals were viewed using a fluorescent laser scanning microscope (40× objective in Leica DMI 3000 microscope).

Quantitative analysis of O_2_^−^ production was performed by using an image analyzer (LEICA Q550 IWB). Three sections per animal were quantified and averaged for each experimental condition. The mean fluorescence densities in the target region were analyzed. Results are expressed as an n-fold increase over the values of the control group in arbitrary units.

### Lipidomic analysis

Methanol:chloroform (1:2) cardiac extracts were divided into two aliquots. One was evaporated to dryness and the pellet was resuspended in 250 µl of acetone:propanol:ethanol (3:4:3) and used to measure triglyceride (TG) content. The other aliquot was evaporated to dryness and the pellet re-suspended in 200 µl methanol:water (9:1) and used to measure phospholipids (PPLs). Extracts were kept at −80°C until analysis.

TG compounds were eluted at a flow rate of 0.4 ml/min using a gradient as follows: initial, 100% A; 3 min, 100% A; 6 min, 98% A; 8 min, 98% A; 9.5 min, 95% A; 11 min, 95% A; 16 min, 100% A, and this was kept isocratic for 2 min to recover initial pressure before next injection. Solvents A and B were methanol:acetonitrile:isopropanol (Met:ACN:IPr 30:30:40, v/v/v) and ACN:IPr (3:7, v/v), respectively, both 0.1% NH_4_OH (25%). To avoid the carry-over, which was calculated to be 12% for unsaturated TGs and 5% for saturated TGs, methanol was injected and an entire chromatographic run was performed, with an additional one performed after five samples were injected. An extract volume of 7.5 µl was injected into chromatographic columns. Mass spectrometric analysis was performed in positive mode (ESI+), using the following parameters: capillary, 0.8 kV; sampling cone, 15 V; source temperature, 90°C; desolvation temperature, 280°C; cone gas, 40 l/h; and desolvation gas, 700 liters/h. Data were acquired with the software Mass Lynx at a rate of 5 scans/s within the range 0-18 min, and m/z 100-1200 Da for the low-energy function and m/z 100-900 Da for the high-energy function (MS^E^ method, trap collision energy 30 V). LC and MS methods were optimized by using the commercial standards TG (18:2/18:2/18:2) and TG (16:0/16:0/16:0). These standards were also used to draw calibration curves for quantification.

PPLs compounds were eluted at a flow rate of 0.35 ml/min using a gradient as follows: initial, 100% A; 1 min, 100% A; 2.5 min, 20% A; 4 min, 20% A; 5.5 min, 0.1% A; 8.0 min, 0.1% A; 10 min, 100% A, and this was kept isocratic for 2 min to recover initial pressure before the following injection. Solvents were (A) methanol:water:formic acid (Met:H_2_O:FA 50:50:0.5, v/v/v) and (B) Met:ACN:FA (59:40:0.5, v/v/v), both with 5 mmol/l ammonium formate. Methanol was injected every five samples and an entire chromatographic run was performed in order to clean the system for possible carry-over (<1%). An extract volume of 7.5 µl was injected. Mass spectrometer parameters were fitted as follows: capillary, 0.9 kV; sampling cone, 18 V; source temperature, 90°C; desolvation temperature, 320°C; cone gas, 45 l/h; and desolvation gas, 900 l/h. Data were acquired with the software MassLynx at a rate of 5 scans/s within the range 0-12 min and 100-1200 Da m/z for the low-energy function, and 50-900 Da m/z for the high-energy function (MS^E^ method, trap collision energy 30 V), with ionization in positive mode (ESI+) for detection of diacylphosphatidylcholines (PCs), ceramides (Cer) and sphingomyelins (SM). External commercial standards PC (10:0/10:0) were used for method optimization and quantification.

Up to three different chromatograms were manually checked for mass spectral peak identification where possible. Within each chromatographic point, m/z values with an intensity ≥700 were checked for this in order to afford a defined chromatographic peak (Extracted Ion Chromatogram, EIC); if positive, the elemental composition tool was then used to determine all the possible chemical compositions (C_n_H_m_O_p_N_s_P_r_S_t_) that were compatible with the isotopic distribution (M, M+1, M+2 and M+3 peaks) of a given m/z value.

Using LipidMaps, Metlin, CheBI, LipidBank and KEGG databases, a particular elemental composition was searched for possible known compounds. Where possible, acyl chains were aimed at being identified by data from the high-energy function (fragmentation). To assess the specific location of each acyl chain at the positions sn-1 or sn-2 of the glycerol backbone is not possible by using this methodology; thus, the most-current structure is indicated. The chromatographic peak area from the EIC of every m/z value detected, whether or not having been identified, was quantified using the QuanLynx application.

Chromatograms and mass spectra of all samples were processed with a MarkerLynx method in order to search for differential features (retention time m/z) amongst sample groups. Five injections of methanol were used as blanks to determine features prone to rejection. Only features that appeared in at least 66% of the samples were accepted. Sets of about 1650 features for data in negative mode and about 2109 features for data in positive mode were detected using the MarkerLynx application. They were checked manually to remove all the features that were present in the blanks. The array resulting from this process, which is comprised of samples and features as independent variables, and the feature signal intensity as dependent variable was submitted to multivariate statistical analysis using the Extended Statistics application that is available with the instrument software; this application is licensed from part of the statistical software SIMCA+ from Umetrics Ltd. (Sweden).

### Cell culture

Cells of the H9c2 rat cardiomyoblast cell line (Merck, Darmstadt, Germany) were maintained in Dulbecco's modified Eagle's medium (DMEM; Merck, Darmstadt, Germany) supplemented with 25 mmol/l glucose, 1 mmol/l pyruvate and 2 mmol/l L-glutamine. Cells were cultured according to the manufacturer′s instructions and were used until passages 20-22. Cells were stimulated with 100, 200 or 300 µmol/l of palmitate – with bovine serum albumin (BSA) as a carrier – conjugated to 10% free fatty acids (FFA)-free BSA for 24 h for the different analyses in order to choose the dose appropriate for performing the experiments. The dose of 200 µmol/l was finally used in all analyses in the presence or absence of MPC (0.01%), which was added before incubation with the palmitic acid.

### Measurements of cellular respiration and estimation of the rate of glycolysis

An XF24-3 extracellular flux analyzer (Seahorse Biosciences, North Billerica, MA) was used to determine the bioenergetic profile of the H9c2 cardiac myoblasts. 40×10^3^ cells/well were seeded in Seahorse XF24 plates and stimulated for 24 h with palmitate-BSA conjugated in 10% FFA-free BSA in the presence or absence of MCP. For the XF24-3 assays, DMEM used initially (see above) was replaced with fresh DMEM supplemented with 5.5 mmol/l glucose, 1 mmol/l pyruvate and 10 mmol/l L-glutamine, stimuli were added again and cells incubated at 37°C in a CO_2_-free incubator for 1 h. Subsequently, the oxygen consumption rate (OCR) and extracellular acidification rate (ECAR), a proxy for lactate production, were recorded to assess the mitochondrial respiratory activity and glycolytic activity, respectively. After four measurements under basal conditions, cells were treated sequentially with 1 µmol/l oligomycin, 0.6 µmol/l carbonyl cyanide p-(trifluoromethoxy)phenylhydrazone (FCCP) and 0.4 µmol/l FCCP with three consecutive determinations under each condition that were averaged during data evaluation. At the end of the run, 1 μmol/l rotenone and 1 µmol/l antimycin A were added to determine the mitochondria-independent oxygen consumption and the value subtracted from all OCR measurements. ATP turnover was estimated from the difference between the basal and the oligomycin-inhibited respiration, and maximum respiratory capacity was the rate in the presence of the uncoupler FCCP. Protein concentration in each well was determined using the BCA method and results were normalized according to protein content. Experiments were repeated four times with similar results.

### Viability assay

H9c2 cell proliferation was evaluated by using the Promega kit (Madison, WI), Cell Titer 96^®^ Aqueous One Solution Cell Proliferation Assay, according to the manufacturer's recommendations. Briefly, cells were seeded in 96-well plates and serum-starved for 24 h. Cells were then stimulated with 100, 200 or 300 µmol/l of palmitate-BSA or 20% of fetal bovine serum (FBS). After 24 h of incubation, formazan product formation was assayed by recording the absorbance at 490 nm in a 96-well plate reader (OD value). Formazan is measured as an assessment of the number of metabolically active cells and expressed in percentages relative to unstimulated cells.

### Fatty acid oxidation assay

H9c2 myoblasts were seeded on six-well plates, grown until semi-confluence and serum-starved overnight. Then, cells were incubated with ^3^H-Palmitate (0.25 µCi/ml) for 1 h at 37°C, and washed three times with 0.5% BSA-PBS to remove any unincorporated and surface-bound fatty acid. Subsequently, cells were pre-treated with 5 µmol/l of triacsin C for 30 min at 37°C and stimulated with 200 µmol/l of palmitate-BSA. After 6 h of incubation at 37°C, DMEM was removed from the plates and total cellular lipids were extracted according to the method of Bligh and Dyer (Bligh and Dyer, 1959). The incorporation of ^3^H-palmitate into total lipids as well as the radioactivity present in the aqueous phase corresponding to ^3^H-soluble metabolites (taken as measure of fatty acid β oxidation) were assayed for radioactivity by liquid scintillation counting.

### Measurement of mitochondrial superoxide anion production and mitochondrial inner transmembrane potential detection

For detection of mitochondrial ion O_2_^−^ production, H9c2 myoblasts were stimulated at 37°C with 200 μmol/l palmitate-BSA in the absence or presence of MCP (0.01%). Thereafter, cells were washed and loaded with 5 μmol/l MitoSOX^TM^ Red for 30 min, at 37°C. The fluorescent signal was analyzed by recording FL2 fluorescence in a Gallius^TM^ flow cytometer (Beckman Coulter).

To evaluate the mitochondrial transmembrane potential (ΔΨm), H9c2 myoblasts were incubated with 4 μmol/l of Rhodamine 123 for 15 min at 37°C. Stained cells were washed with serum-free medium and stimulated with the indicated doses with palmitate-BSA for 24 h at 37°C. After treatment, cells were washed with PBS and changes in fluorescence were monitored using flow cytometry analysis. In some experiments, before incubation with 200 µmol/l of palmitate-BSA, H9c2 cells were pretreated for 30 min with 3 µmol/l of Triacsin C, an inhibitor of long fatty acid acyl-CoA synthetase (Sigma, St Louis, MO) or with the indicated dose of MCP. Experiments were repeated at least three times. Cells were also visualized on a Leica TCS SP5 X confocal microscope with a ×40 objective.

### Measurement of mitochondrial cardiolipin by using NAO

10-N-nonyl-Acridin Orange (NAO; Invitrogen, Carlsbad, CA) is a fluorochrome that binds to intact mitochondrial cardiolipin and it is independent of the mitochondrial membrane potential over a physiologically relevant range (Maftah et al., 1989; Petit et al., 1992). Decreases in the fluorescence of NAO in cells have been reported to reflect the peroxidation of intracellular cardiolipin because the dye loses its affinity for peroxidized cardiolipin (Nomura et al., 2000).

H9c2 cells were treated with 100, 200 or 300 µmol/l of palmitic acid conjugated to BSA for 24 h at 37°C. Afterwards, cells were stained with 5 μmol/l of NAO for 15 min at 37°C in dark. Cells were then washed with PBS and NAO fluorescence intensity was analyzed by recording FL1 fluorescence in a GalliusTM flow cytometrer (Beckman Coulter). Cells were also visualized on a Leica TCS SP5 X confocal microscope with a ×40 objective. The cells were excited using 488 nm and emission of NAO was measured beyond 585 nm. Nuclei of cells were stained with DAPI as a counter stain. Experiments were repeated at least three times.

### Statistical analysis

Continuous variables are expressed as mean±s.e.m. Normality of distributions was verified by means of the Kolmogorov–Smirnov test. One-way ANOVA was used and followed by Tukey test. Either Pearson or Spearman correlation analysis was used to examine association among different variables according to whether they are normally distributed. A value of *P*<0.05 was used as the cutoff value for defining statistical significance. Data analysis was performed using the statistical program SPSS version 22.0 (SPSS Inc, Chicago, IL).

## Supplementary Material

Supplementary information
