# Supplementary material for: Inhibition of galectin-3 ameliorates the consequences of cardiac lipotoxicity in a rat model of diet-induced obesity
Source: Dis Model Mech. 2018 Feb 1;11(2):dmm032086. doi: 10.1242/dmm.032086 (PMC5894945; doi:10.1242/dmm.032086)
Supplement: Supplementary information [file dmm-11-032086-s1.pdf]

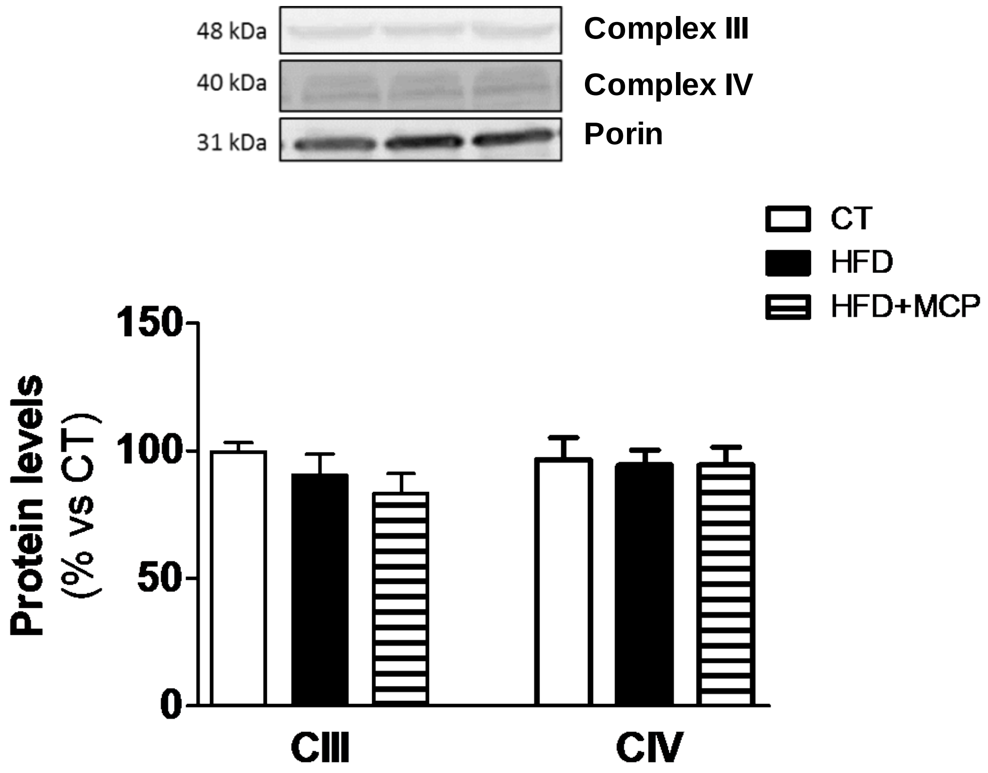

Figure S1. Impact of Gal-3inhibition on protein in heart from control and obese rats. Heart from rats fed a standard diet (CT) or a high fat diet (HFD) treated with vehicle or with the inhibitor of Gal-3 activity (Modified citrus pectin; MCP; 100 mg/Kg/day) were analyzed. Protein expression of subunits 2 and 1 from, respectively, mitochondrial complexes III and IV are presented. Bar graphs represent the mean ± SEM of 6-8 animals normalized to porin.

Disease Models & Mechanisms • Supplementary information

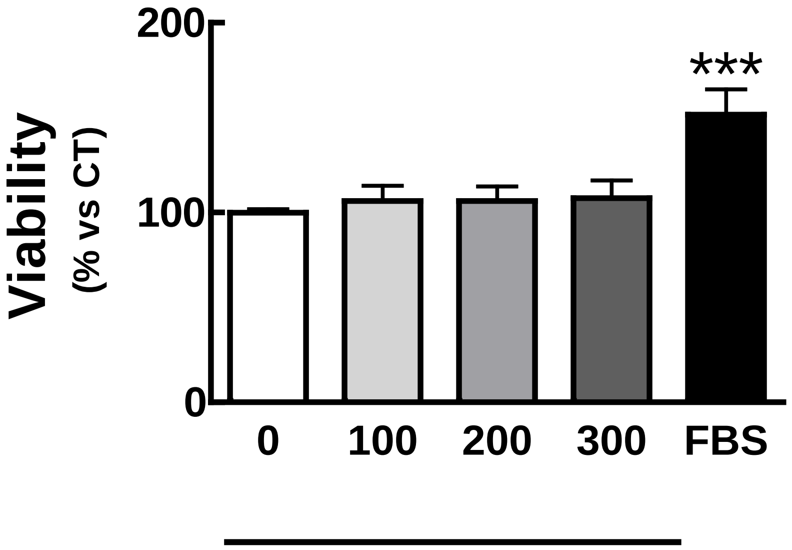

Figure S2. Effects of palmitic acid on the viability in H9c2 cells. Cardiac myoblasts were stimulated with palmitic acid (100-300 µmol/L) or 20 % of fetal bovine serum (FBS) for 24 hours. Viability was determined by an MTT assay. Data are expressed as percent of unstimulated cells. Values are mean±SEM of three assays. ***p<0.001 *vs.* vehicle treated cells.

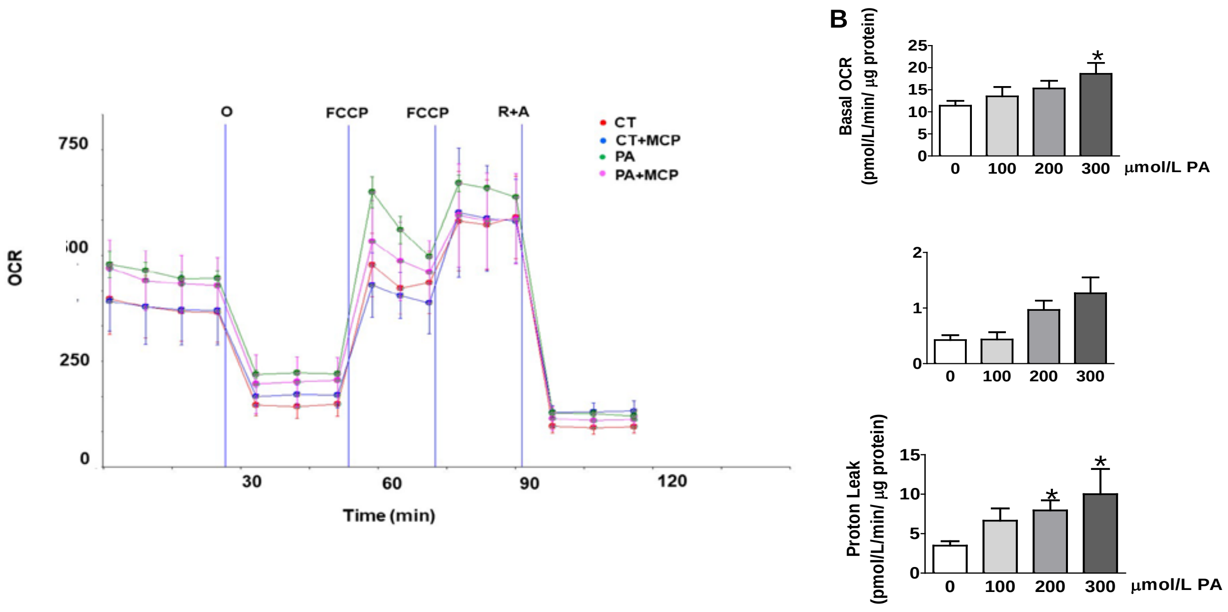

Figure S3. Effects of palmitic acid on mitochondrial function and glycolysis in H9c2 cells. (A) Representative mitochondrial respiratory profile from a XF Mitochondrial Stress Test. Oxygen consumption rate (OCR) was measured under basal conditions followed by the sequential addition (vertical lines) of oligomycin (O; 1 µmol/L); FCCP carbonyl cyanide p-(trifluoromethoxy) phenylhydrazone (0.6 µmol/L), FCCP (0.4 µmol/L); R+A, 1 µM rotenone plus 1 µmol/L antimycin A as indicated. H9c2 myoblasts were treated for 24 hours with palmitic acid (PA; 200 µmol/L) in the presence of absence inhibitor of Gal-3 activity (Modified citrus pectin; MCP; 0.01%). Each data point represents an OCR measurement as mean ± SEM (n = 5). (B) Basal respiration expressed as oxygen consumption rate (OCR), (C) Basal glycolysis expressed as extracellular acidification rate (ECAR), (D) proton leak respiration expressed as OCR in cardiac myoblasts treated for 24 hours with palmitic acid (PA;100-300 µmol/L). Bar graphs represent the mean ± SEM of 4 assays. *$p<0.05$ vehicle treated cells (CT).

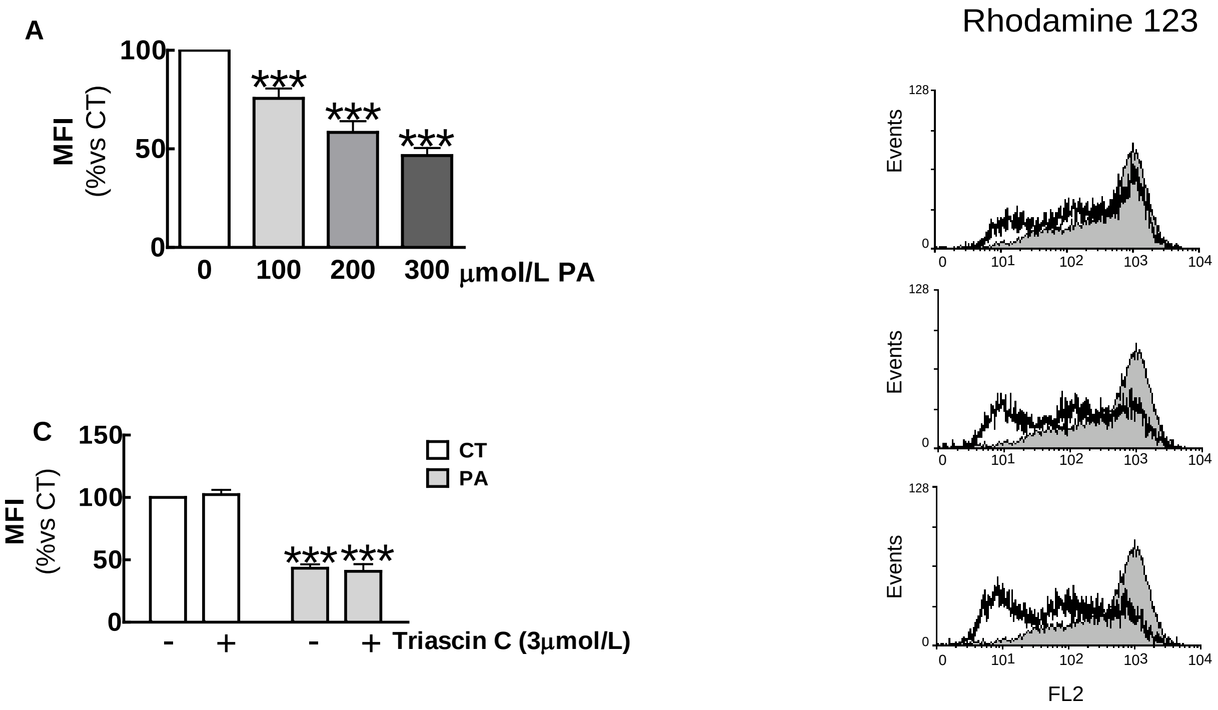

Figure S4. Effects of palmitic acid on mitochondrial membrane potential in H9c2 cells. (A) Quantification of flow cytometry analysis of mitochondrial membrane potential in cardiac myoblasts stained with Rhodamine 123 treated for 24 hours with palmitic acid (PA; 100-300 μmol/L) and expressed as mean fluorescence intensity (MFI), (B) Representative histogram, untreated cells (solid curves) were compared with stimulated cells (open curves), (C) Flow cytometry analysis of mitochondrial membrane potential in cardiac myoblasts stained with Rhodamine 123 treated for 24 hours with palmitic acid (200 μmol/L) in the presence of absence of the inhibitor of long fatty acyl CoA synthetase, Triascin C (3 μmol/L) and expressed a MFI. Bar graphs represent the mean ± SEM of 3 assays. ***p<0.001 vehicle treated cell.

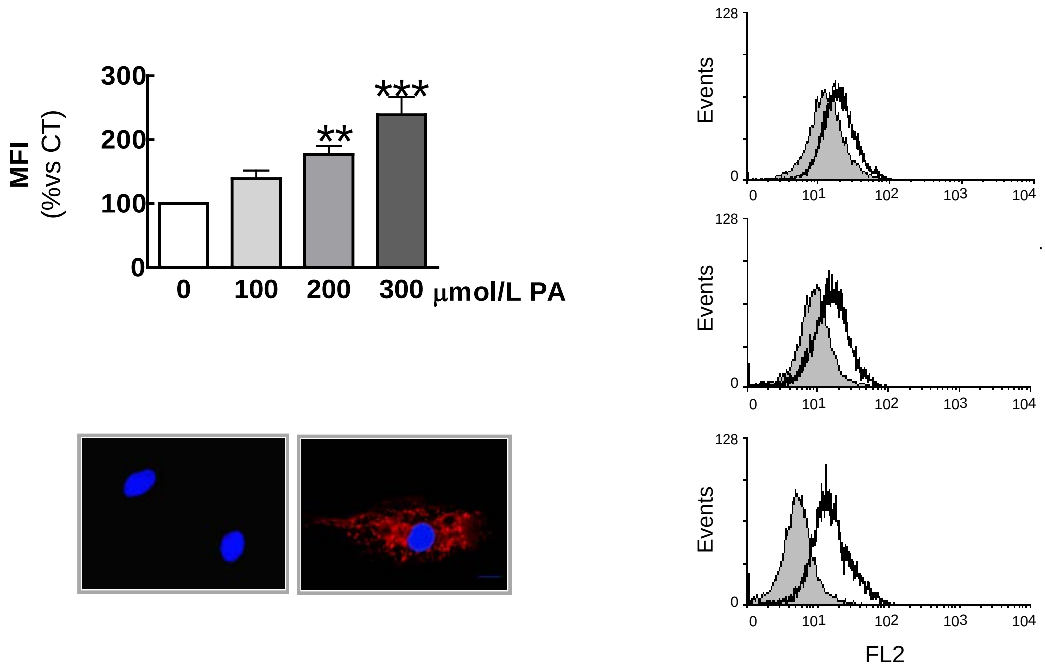

Figure S5. Effects of palmitic acid on ROS production in H9c2 cells. (A) Quantification of flow cytometry analysis of mitochondrial superoxide anions in cardiac myoblasts labeled with MitoSox and treated for 24 hours with palmitic acid (PA; 100-300 µmol/L) and expressed as mean fluorescence intensity (MFI), (B) Representative histogram, untreated cells (solid curves) were compared with stimulated cells (open curves), (C) Representative microphotographs showing H9c2 cells labeled with MitoSox. Nuclei of cells were co-stained with DAPI. Bar graphs represent the mean ± SEM of 3 assays. ** p<0.01; ***p<0.001 vehicle treated cell.

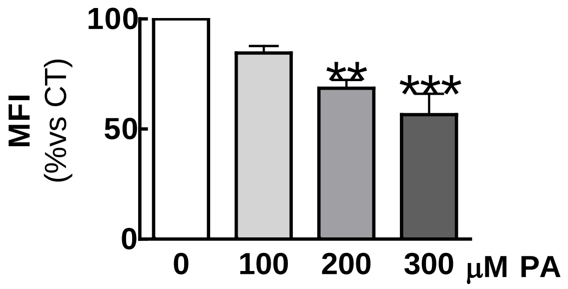

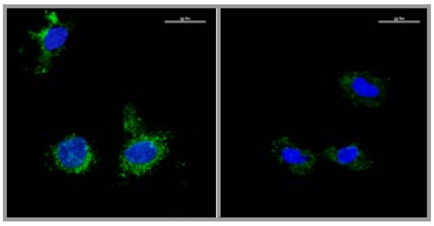

Figure S6. Effects of palmitic acid on cardiolipin oxidation in H9c2 cells. (A) Quantification of flow cytometry analysis of cadiolipin oxidation levels in cardiac myoblasts stained with 10-Nony acridine orange treated for 24 hours with palmitic acid (PA;100-300 µmol/L) and expressed as mean fluorescence intensity (MFI). (B) Representative microphotographs showing H9c2 cells labeled with 10-Nony acridine orange. Nuclei of cells were co-stained with DAPI. Representative microphotographies. Bar graphs represent the mean ± SEM of 3 assays. ** $p<0.01$; ***$p<0.001$ vehicle treated cell.

Disease Models & Mechanisms • Supplementary information

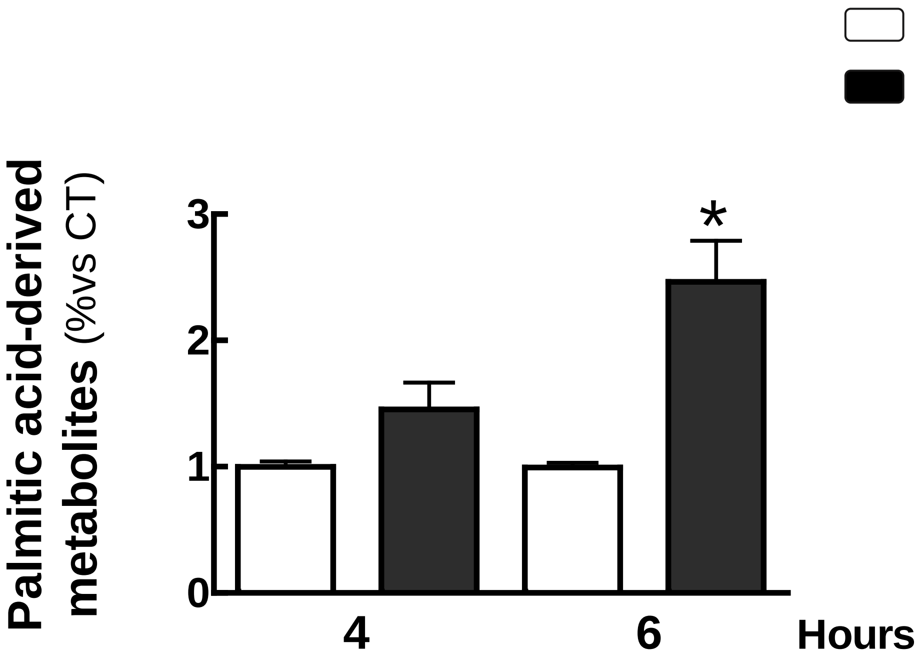

Figure S7. Effects of palmitic acid on β-oxidation in H9c2 cells. Quantification of on β-oxidation in cardiac myoblasts treated for 4 or 6 hours with palmitic acid (PA; 200 µmol/L) and expressed as palmitic acid hydrosoluble derived metabolites. Bar graphs represent the mean ± SEM of 3 assays. * $p<0.05$ vehicle treated cell.

Disease Models & Mechanisms • Supplementary information
